# Bacterial Sacroiliitis Probably Induced by Lumbar Epidural Analgesia

**DOI:** 10.1080/10647440300025506

**Published:** 2003

**Authors:** Shimon Edelstein, Yeouda Edoute

**Affiliations:** Department of Internal Medicine C Rambam Medical Center and the Bruce Rappaport Faculty of Medicine Technion-Israel Institute of Technology PO Box 9602 Haifa 31096 Israel

## Abstract

*Background:* Properly administered, lumbar epidural analgesia provides adequate pain relief during labor and delivery, and is considered to be a safe procedure with limited complications. The prevalence of infection after lumbar epidural analgesia is negligible.

*Introduction:*  Infection of the sacroiliac joint, although very close to the pucture area, has never been reported as a procedure complication.

Case: In this report, we describe a patient who experienced bacterial sacroiliitis a few days after lumbar epidural analgesia for labor. No portal of entry was identified, and we evoked a new potential risk factor that has never been proposed before, namely lumbar epidural analgesia.

*Conclusion:*  Sacroiliitis must be considered as a rare but serious complication of lumbar epidural analgesia.


*Infect Dis Obstet Gynecol 2003;11:105–108*

### Bacterial sacroiliitis probably induced by lumbar epidural analgesia

**Shimon Edelstein and Yeouda Edoute**

*Department of Internal Medicine C, Rambam Medical Center and the Bruce Rappaport Faculty of Medicine, Technion-Israel Institute of Technology, Haifa, Israel*

***Background:*** Properly administered, lumbar epidural analgesia provides adequate pain relief during labor and delivery, and is considered to be a safe procedure with limited complications. The prevalence of infection after lumbar epidural analgesia is negligible.
***Introduction:*** Infection of the sacroiliac joint, although very close to the pucture area, has never been reported as a procedure complication.
***Case:*** In this report, we describe a patient who experienced bacterial sacroiliitis a few days after lumbar epidural analgesia for labor. No portal of entry was identified, and we evoked a new potential risk factor that has never been proposed before, namely lumbar epidural analgesia.
***Conclusion:*** Sacroiliitis must be considered as a rare but serious complication of lumbar epidural analgesia.

Key words: COMPLICATION; OSTEOMYELITIS; BONE SCAN; LUMBAR PUNCTURE

### CASE

A 25-year-old woman delivered a healthy baby under lumbar epidural analgesia and was discharged home in good condition and with a normal temperature. The delivery was undertaken at a different hospital, but according to the description on the discharge file, both the delivery and the lumbar epidural analgesia were normal without the need for instrumentation, evidence of soft tissue trauma or vaginal wall tears or any other complication. No injections were given apart from the epidural analgesia. According to the patient's chart the lumbar epidural analgesia was described as 'usual with no complications', at L3-L4 intervertebral space, using an 18-gauge needle to penetrate and bupivacaine as an analgesic medication. Five days after discharge, the patient experienced fever (39.5°C) and chills, malaise and right buttock pain. Over the next day the pain increased in intensity until she was unable to bear weight on her right leg. She was then hospitalized for evaluation. Her past medical history was unremarkable except for allergy to penicillin, manifested by skin rash. She denied a history of trauma or drug use.

On physical examination, she had a temperature of 40°C, pulse rate of 116 beats/minute, respiratory rate of 24 breaths/minute, and blood pressure of 90/60 mmHg. She had severe pain over the right sacroiliac joint posteriorly, and pelvic compression elicited severe pain in the right sacroiliac joint. Gynecological and neurological examinations were unremarkable.

The patient's laboratory test results were significant for an elevated erythrocyte sedimentation rate (ESR) of 115 mm/hour and leukocytosis of 11 100 cells/$mm^3$ with left shift. Three bottles of

Correspondence to: Shimon Edelstein, MD, Department of Internal Medicine C, Rambam Medical Center, PO Box 9602, 31096 Haifa, Israel. Email: s_edelstein@rambam.health.gov.il

© 2003 The Parthenon Publishing Group 

blood cultures were obtained in the initial hours after arrival, prior to antibiotic initiation. The patient refused to undergo sacroiliac joint aspiration, even though the staff made an effort to explain the importance of pathogen isolation in order to narrow the antibiotic spectrum and minimize the side-effects. The blood cultures that were taken prior to and during the antibiotic treatment demonstrated no growth. Chest X-ray, plain roentgenograms and computed tomography (CT) of the pelvis and lumbosacral spine were normal on admission. Technetium-99 methylene diphosphonate (Tc-99m MDP) bone scintigraphy demonstrated mildly increased uptake in the right sacroiliac region (Figure 1). Gallium scintigraphy demonstrated increased uptake of Ga-67 citrate in the right sacroiliac region. The uptake was greater with regard to area and intensity compared with the bone scan (Figure 2). These findings suggested a diagnosis of acute infectious right sacroiliitis.

Septic arthritis of the sacroiliac joint was suspected, and a regimen of intravenous vancomycin 1 g every 12 hours and gentamicin 80 mg every 8 hours was administered. Within 48 hours the patient's fever responded, and after 10 days she noticed decreased pain. On day 12 gentamicin was stopped because of nephrotoxicity despite desirable gentamicin levels (pre- and post-administration) on hospitalization days 3 and 7. After 17 days of therapy the patient was

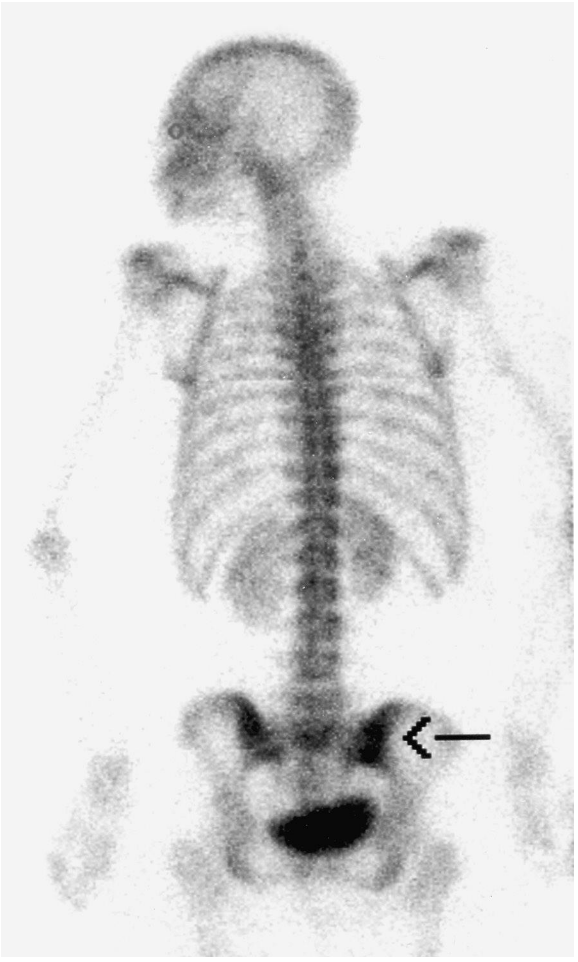

**Figure 1** Bone scintigraphy (posterior view) 2 hours after intravenous injection of 24 mCi of Tc99m-MDP demonstrates mildly increased uptake of the tracer in the right sacroiliac region (arrow)

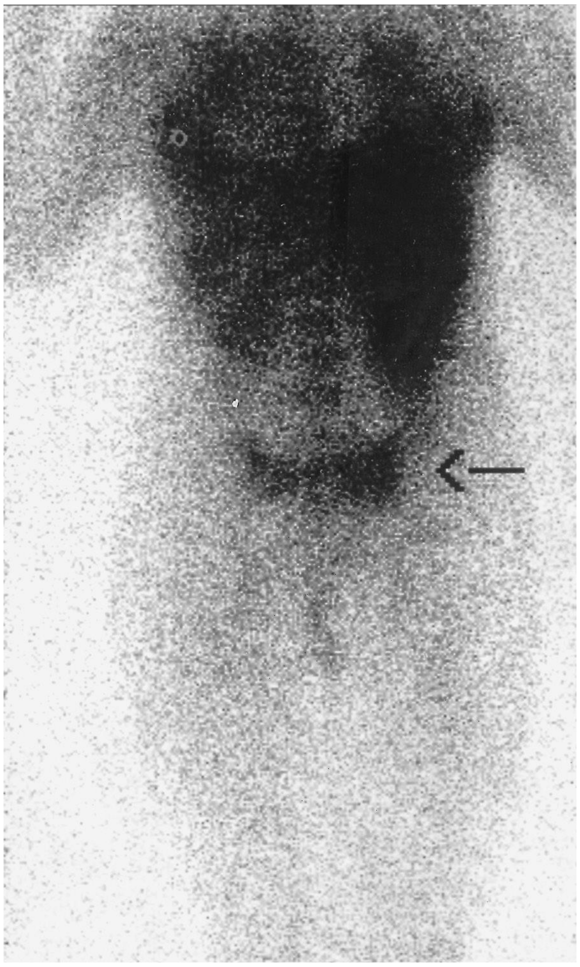

**Figure 2** Gallium scintigraphy (posterior view) 24 hours after intravenous injection of Ga-67 citrate demonstrating increased uptake of the tracer in the right sacroiliac region (arrow). The uptake was greater with regard to intensity and area compared with the bone scan. The findings are consistent with right sacroiliitis ambulatory, and she continued to show steady improvement thereafter. No bone scan or gallium scan was repeated, since the patient felt much better and refused to undergo further examinations. She was discharged home after 28 days of hospitalization, and a regimen of oral fucidinic acid and ofloxacin was recommended for 2 weeks. Eight weeks after discharge the patient had recovered completely and her ESR had returned to normal.

### DISCUSSION

Bacterial sacroiliitis is a rare infection[1–12], and the diagnosis is frequently missed unless the physician is familiar with the disease. A delay in the diagnosis may be associated with marked toxemia, and may necessitate surgical drainage of the septic joint. It may rarely even lead to death. The patient's diagnosis was based on the following criteria: first, clinical symptoms of acute bacterial infection (severe continuous pain exacerbated by weight-bearing or any attempt to move the sacroiliac joint, high ESR, and leukocytosis with a left shift); secondly, bone and gallium scintigraphy demonstrating intense focal uptake in the right sacroiliac joint; and thirdly, an excellent clinical response to antibiotic therapy.

In patients with bacterial sacroiliitis, *Staphylococcus aureus* and *Streptococcus pneumoniae* are the most frequently isolated organisms[1,6,8], while *Pseudomonas aeruginosa* is commonly encountered in intravenous drug abusers[4,8,10,11,13]. Blood cultures are positive in only one-third to two-thirds of these patients[2,5,8,10]. Plain roentgenograms and CT scans of the pelvis are usually normal at presentation[1,4,8], as was the case in this patient. The earliest changes on the plain films occur 2 weeks after the onset of symptoms, and consist of blurring and erosions of the margins of the sacroiliac joint, with widening of the joint space[1,5].

Scintigraphy is more sensitive for detecting early bacterial sacroiliitis[4,6,10,13], and can demonstrate abnormalities in the joints as early as 2–6 days into the illness[4,8]. The Tc-99m MDP bone scintigraphy and Ga-67 scintigraphy findings in the present patient suggested a diagnosis of acute infectious right sacroiliitis. Needle aspiration of the suspected infected bone is highly recommended, as a wide spectrum of pathogens may infect the bone and thus a wide spectrum of antibiotic coverage must be given, if no pathogen is isolated. Since a specific pathogen was not isolated in this case, empirical broad-spectrum antibiotic therapy with vancomycin and gentamicin was directed toward *Staphylococcus aureus* and Gram-negative bacteria, and resulted in complete recovery 8 weeks after discharge of the patient.

Lumbar epidural analgesia is considered to be a safe procedure with limited complications[14]. In a review of the maternal complications in a consecutive series of over 27 000 instances of lumbar epidural analgesia used during labor, Crawford[15] reported only one case of infection, in which lumbar epidural analgesia given to a woman was complicated by occult streptococcal bacteremia. Source investigation yielded a small infected hematoma in the epidural space[15]. Bacterial sacroiliitis usually occurs in patients with underlying conditions such as trauma, a history of intravenous drug abuse or a concurrent source of bacterial infection[6,10–13].

In the present case, the presentation of bacterial sacroiliitis 5 days after delivery under lumbar epidural analgesia and the absence of an underlying condition suggest that in this woman the bacterial sacroiliitis could be attributed to the lumbar epidural analgesia procedure during her labor. Neither delivery complications nor any intramuscular injections were noted on her chart. The patient denied any local pain or trauma prior to her last hospitalization. Bacteria may be introduced to the joint through local invasion (through ruptured skin – lumbar epidural analgesia) or hematogeneous seeding during the peri-procedure period. There was no clue to support the possibility of hematogenic seeding, such as fever, chills, systemic inflammatory response syndrome (SIRS) or sepsis, which would be expected to be present if the mechanism was hematogenic. Although bacterial sacroiliitis following lumbar epidural analgesia has not been previously reported in the literature, the proximity and timing, as well as the absence of any other known risk factor, all suggest the possibility of lumbar epidural analgesia as a portal of entry. We consider it very important to report this finding, since it is clear that bacterial sacroiliitis should be considered as a possible serious complication of this procedure. Further reports are needed to establish the connection between this proposed causative pathogenesis of lumbar epidural analgesia and bacterial sacroiliitis.

### REFERENCES

1. Delbarre F, Rondier J, Delrieu F, *et al*. Pyogenic infection of the sacroiliac joint. Report of thirteen cases. *J Bone Joint Surg Am* 1975;57:819–25
2. Coy JT III, Wolf CR, Brower TD, *et al*. Pyogenic arthritis of the sacroiliac joint. Long-term follow-up. *J Bone Joint Surg Am* 1976;58:845–9
3. Dunn EJ, Bryan DM, Nugent JT, *et al*. Pyogenic infections of the sacroiliac joint. *Clin Orthop* 1976;118:113–17
4. Gordon G, Kabins SA. Pyogenic sacroiliitis. *Am J Med* 1980;69:50–6
5. Longoria RR, Carpenter JL. Anaerobic pyogenic sacroiliitis. *South Med J* 1983;76:649–51
6. Oka M, Mottonen T. Septic sacroiliitis. *J Rheumatol* 1983;10:475–8
7. Jajic I, Furst Z, Kraij K, *et al*. Septic sacroiliitis. An analysis of 14 patients. *Acta Orthop Scand* 1983;54:210–11
8. Kerr R. Pyogenic sacroiliitis. *Orthopedics* 1985; 8:1028–34
9. Shanahan MDG, Ackroyd CE. Pyogenic infection of the sacroiliac joint. A report of 11 cases. *J Bone Joint Surg Br* 1985;67:605–8
10. Shapiro SK, See CE. Pyogenic sacroiliitis. *Minn Med* 1986;69:201–4
11. Guyot DR, Manoli A II, Kling GA. Pyogenic sacroiliitis in i.v. drug abusers. *Am J Roentgenol* 1987;149:1209–11
12. Vyskocil JJ, McIlroy MA, Brennan TA, *et al*. Pyogenic infection of the sacroiliac joint. Case reports and review of the literature. *Medicine (Baltimore)* 1991;70:188–97
13. Abbott GT, Carty H. Pyogenic sacroiliitis, the missed diagnosis? *Br J Radiol* 1993;66:120–2
14. Lurie S, Priscu V. Update on epidural analgesia during labor and delivery. *Eur J Obstet Gynecol Reprod Biol* 1993;49:147–53
15. Crawford JS. Some maternal complications of epidural analgesia for labour. *Anaesthesia* 1985; 40:1219–25

Received 10/22/02; Accepted 04/08/03
